# Inflammatory stimuli and hypoxia on atherosclerotic plaque thrombogenicity: Linking macrophage tissue factor and glycolysis

**DOI:** 10.1371/journal.pone.0316474

**Published:** 2025-03-04

**Authors:** Kazunari Maekawa, Eriko Nakamura, Yoichi Saito, Yunosuke Matsuura, Toshihiro Gi, Kensaku Nishihira, Nobuyuki Oguri, Sayaka Moriguchi-Goto, Yuichiro Sato, Kinta Hatakeyama, Yoshisato Shibata, Yoshihiro Komohara, Koichi Kaikita, Yujiro Asada, Atsushi Yamashita

**Affiliations:** 1 Department of Pathology, Faculty of Medicine, University of Miyazaki, Miyazaki, Miyazaki, Japan; 2 Bioengineering Lab, Faculty of Advanced Science and Technology, Kumamoto University, Kumamoto, Kumamoto, Japan; 3 Division of Cardiovascular Medicine and Nephrology, Department of Internal Medicine, Faculty of Medicine, University of Miyazaki, Miyazaki, Miyazaki, Japan; 4 Department of Cardiology, Miyazaki Medical Association Hospital, Miyazaki, Miyazaki, Japan; 5 Department of Pathology, National Cerebral and Cardiovascular Center Hospital, Suita, Osaka, Japan; 6 Department of Cell Pathology, Graduate School of Medical Sciences, Faculty of Life Sciences, Kumamoto University, Kumamoto, Kumamoto, Japan; 7 Department of Diagnostic Pathology, Miyazaki Medical Association Hospital, Miyazaki, Miyazaki, Japan; Kyoto University Graduate School of Medicine Faculty of Medicine: Kyoto Daigaku Daigakuin Igaku Kenkyuka Igakubu, JAPAN

## Abstract

**Background:**

The thrombogenic potential of cells within atherosclerotic plaques is critical in the formation of a coronary thrombus. We hypothesized that a combination of inflammatory and hypoxic stimuli enhances tissue factor (TF) expression and glycolysis in cells in atherosclerotic plaques and contributes to coronary thrombus formation.

**Aims:**

To identify TF- and hexokinase (HK)-II-expressing cells in coronary atherosclerotic plaques and thrombi and determine the effects of combined inflammatory and hypoxic stimuli and glycolysis on TF expression in peripheral blood mononuclear cell-derived macrophages.

**Methods:**

We immunohistochemically assessed TF and HK-II expression in stable (n =  20) and unstable (n =  24) human coronary plaques and aspirated acute coronary thrombi (n =  15). The macrophages were stimulated with tumor necrosis factor-α, interferon-γ, or interleukin-10 under normoxic (21% O_2_) or hypoxic (1% O_2_) conditions, and TF expression was assessed.

**Results:**

TF and HK-II expression were increased in unstable plaques compared with stable plaques. The number of CD68- and HK-II-immunopositive cells positively correlated with the number of TF-immunopositive cells. TF- and HK-II-expressing macrophages, which expressed M1- or M2-like markers, were involved in platelet-fibrin thrombus formation in ruptured plaques. The combination of inflammatory and hypoxic conditions additively augmented TF expression and procoagulant activity in the cultured macrophages. Inhibition of glycolysis with 2-deoxyglucose reduced the augmented TF expression and procoagulant activity.

**Conclusion:**

Combined inflammatory and hypoxic conditions in atherosclerotic plaques may markedly enhance procoagulant activity in macrophages and contribute to coronary thrombus formation following plaque disruption. Macrophage TF expression may be associated with glycolysis.

## Introduction

Cardiovascular diseases are the leading causes of disease burden worldwide, with the total number of disability-adjusted life years and deaths attributable to ischemic heart disease continuing to increase globally [1]. Acute coronary syndrome (ACS) is an acute form of ischemic heart disease caused by coronary plaque disruption and subsequent platelet- and fibrin-rich thrombus formation [2]. The most common morphological entity responsible for plaque disruption is plaque rupture, while less common entities include plaque erosion and calcified nodules [3]. Rupture-prone atherosclerotic lesions harbor lipid-rich necrotic cores, which contain large amounts of tissue factor (TF), an initiator of blood coagulation [4,5]. Plaque disruption exposes the TF-rich necrotic core to the bloodstream, which is followed by the initiation of the coagulation cascade as well as platelet aggregation. Plaque-derived TF contributes to platelet-fibrin thrombus formation in atherosclerotic lesions via excess thrombin generation [6]. The size of coronary thrombi is correlated with the length of plaque disruption and TF-immunopositive areas in the plaques [7]. However, pathological evidence showing the presence of TF-expressing cells in coronary thrombi remains lacking.

Macrophages play the most important role in the development of atherosclerotic cardiovascular diseases, and macrophage phenotypes are related to the progression or regression of atherosclerosis [8]. Indeed, Kaikita et al. reported that TF expression in macrophages was higher in plaques of unstable angina than in those of stable angina [9]. These findings highlight the importance of understanding the mechanisms underlying TF overexpression in atherosclerotic plaques.

TF expression in human macrophages is enhanced by Th1 cytokines (interferon [IFN]-γ and tumor necrosis factor [TNF]-α), Th2 cytokines (interleukin [IL]-4 and IL-13), lipopolysaccharide and IFN-γ, oxidative stress, and hypoxia [10–13]. In contrast, IL-10 negatively regulates lipopolysaccharide-induced TF expression in macrophages [14].

Rupture-prone atherosclerotic lesions are characterized by the presence of abundant inflammatory cells and a large necrotic core. Thus, several factors may simultaneously affect TF expression in rupture-prone plaques. Okuyama et al. observed a positive relationship between coronary artery thrombus size and the expression of TF and hexokinase (HK)-II in coronary artery plaques [7]. Hypoxic condition in rabbit atherosclerotic lesions enhances HK-II expression and nuclear localization of hypoxia-inducible factor-1a, increasing glycolysis [15]. However, whether glycolysis enhances TF expression in macrophages remains unclear. In addition, although hypoxia augments TF expression in cultured macrophages, the extent of the upregulation was, at most, ~ 1.5-fold [13].

Therefore, we hypothesized that combining inflammatory and hypoxic stimuli has a crucial effect on enhancing TF expression in cells within atherosclerotic plaques. In this study, we aimed to identify TF- and HK-II-expressing cells in human coronary atherosclerotic plaques and thrombi and determine the effects of combined inflammatory and hypoxic stimuli on TF expression in peripheral blood mononuclear cell (PBMC)-derived macrophages.

## Materials and methods

### Pathological and immunohistochemical analyses of coronary arteries from autopsy cases

The numbers of immunopositive cells per mm^2^ in whole intimal lesions were compared between the stable and vulnerable lesions to evaluate immunopositive cells for hexokinase (HK)-II, TF, CD68, CD3, matrix metalloproteinase 9 (MMP9), inducible nitric oxide synthase (iNOS), mannose receptor C-1 (MRC-1), CD163, and the number of CD31-immunopositive microvessels. We examined 12 autopsy cases with and without ischemic heart disease; those were performed from January 1, 2000 to December 31, 2015.

Clinical data were obtained from autopsy records. These patients had not undergone coronary interventions or coronary surgery. The mean age of the 12 patients was 72 years (range, 50–91 years). Family history of ischemic heart disease, smoking habit, obesity, hypertension, dyslipidemia, and diabetes were reported in 1 (8%), 8 (67%), 4 (14%), 9 (75%), 2 (17%), and 7 (58%) patients, respectively. Seven of the 12 patients had autopsy-confirmed acute myocardial infarction, and the median duration after the onset of acute myocardial infarction was 11 d (range, 0–30 d). Formalin-fixed, paraffin-embedded tissue sections were processed for pathological analyses. Forty-four coronary artery lesions from autopsy cases were categorized as either stable lesions (American Heart Association [AHA] type I, II, III, IV, or V without a thin fibrous cap) or vulnerable lesions (AHA type V with a thin fibrous cap or AHA type VI) [16].

We used the following antibodies for immunohistochemical analyses: mouse monoclonal anti-HK-II antibody (Abcam, Cambridge, UK), mouse monoclonal anti-TF antibody, H-9, (Santa Cruz Biotechnology, Inc., Dallas, TX, US), mouse monoclonal anti-CD68 antibody, PG-M1 (Agilent, Santa Clara, CA, US), mouse monoclonal anti-CD3 antibody (F7.2.38) (Abcam), mouse monoclonal anti-CD31 antibody (Agilent), mouse monoclonal anti-alpha smooth muscle actin (SMA) antibody (1A4, Thermo Fisher Scientific, Waltham, MA, USA), mouse monoclonal anti-MMP9 antibody (Daiichi Fine Chemical Co., Ltd., Takaoka, Japan), rabbit polyclonal anti-iNOS (a marker of M1-like macrophage) antibody (Novus, Littleton, CO, USA), mouse monoclonal anti-MRC-1 (a marker of M2-like macrophage) antibody (5C11, LifeSpan Niosciences, Seattle, WA, USA), and rabbit monoclonal anti-CD163 (a marker of M2-like macrophage) antibody (EPR19518, Abcam). Negative controls were processed using immunoglobulin G from nonimmune mice (Jackson ImmunoResearch, Baltimore, MA, USA) or nonimmune rabbit (Affinity Biologicals Inc., Hamilton, ON, Canada).

Sections were visualized using the Envision system (Agilent, Santa Clara, CA, US) and 3,3′-diaminobenzidine. Nuclei were counterstained with Meyer’s hematoxylin. Immunopositive cell counts for CD68, HK-II, CD3, TF, MMP-9, iNOS, MRC-1, CD163, and the number of CD31-immunopositive microvessels were analyzed under a 10X objective lens in the atherosclerotic intimal area on every coronary section. The number of immunopositive cells per mm^2^ area was compared between the stable and vulnerable lesions.

We also determined whether coronary calcification affected plaque vulnerability and TF and HK-II expression in the presence or absence of calcification pathology. Coronary calcification was categorized into microcalcification/punctate, fragment, sheet, or nodular calcification, as previously described [17].

This study was approved by the Research Ethics Committee of the University of Miyazaki (2015-186). The ethics committee waived the requirement for informed consent. All studies involving human subjects were performed in accordance with the Declaration of Helsinki.

### Pathological analysis of aspirated coronary thrombus from ACS patients

Acute coronary thrombi in patients with ACS were aspirated using a thrombus aspiration catheter (Thrombuser; Kaneka Medix Co., Osaka, Japan) at the start of percutaneous coronary intervention. The aspirated thrombi were formalin-fixed and paraffin-embedded for pathological examination. We used samples from 15 patients from January 1, 2008 to December 31, 2016; the samples included both thrombus and atheromatous plaque components. The mean age of the 15 patients (2 females and 13 males) was 64 years (range: 53–85 years).

Family history of ischemic heart disease, smoking habit, obesity, hypertension, dyslipidemia, and diabetes were reported in 3 (20%), 11 (73%), 7 (47%), 9 (60%), 8 (53%), and in 3 (20%) patients, respectively. We immunohistochemically examined the presence or absence of macrophages (CD68, PGM-1, Agilent), smooth muscle cells (aSMA, 1A4; Thermo Fisher Scientific), endothelial cells (CD31; Agilent), fibrin (Accurate Chemical & Scientific Corp., Westbury, NY, USA), platelet (glycoprotein (GP) IIb/IIIa (Affinity Biologicals Inc., Ancaster, CA, USA), and expression of TF (H-9; Santa Cruz Biotechnology), HK-II (Abcam), iNOS (Novus), mouse monoclonal MRC-1 (5C11, LifeSpan Niosciences), and CD163 (EPR19518, Abcam) in the coronary thrombus with ruptured plaque. The Research Ethics Committee of the University of Miyazaki approved this study (O-0224). Ethics committee waived the requirement for informed consent.

### Isolation of human peripheral blood mononuclear cells, macrophage differentiation, and cell culture experiment

Human PBMCs were obtained from healthy adult volunteers who were recruited from February 8, 2018 to February 28, 2022. PBMCs were isolated using SepMate-50 (STEMCELL, Vancouver, BC, Canada) and Ficoll-Paque PREMIUM (GE Healthcare, Chicago, IL, US). Cells were cultured in RPMI-1640 (Dainippon Sumitomo Pharm, Osaka, Japan) supplemented with 10% heat-inactivated human plasma (healthy adult volunteers) and ZellShield (Minerva Biolabs, Berlin, Germany). The PBMCs were incubated for 24 h and then washed with phosphate-buffered saline (PBS) to remove floating cells, including lymphocytes, platelets, and contaminant red cells. The attached monocytes remained at the base of the culture dishes [18]. The monocytes were differentiated into macrophages by 6-d incubation in culture media supplemented with 100 ng/mL macrophage colony-stimulating factor (Pepro Tec, Cranbury, NJ, USA) and 2 ng/mL granulocyte-macrophage colony-stimulating factor (GM-CSF; Pepro Tec, Cranbury, NJ, USA). The cells were cultured with TNF-α (10 ng/mL, Sigma-Aldrich Merck, Darmstadt, Germany) and INF-γ (20 ng/mL, R & D Systems, Minneapolis, MN, USA) or IL-10 (10 ng/mL, Pepro Tec, Cranbury, NJ, USA) as previously described [7,19], and/or hypoxia (1% O_2_) for 4 or 24 h to analyze TF and HK-II mRNA or protein expression and lactate production [13], respectively. Glycolysis was inhibited by adding 5 mM of 2-deoxy-d-glucose (2-DG; Sigma, St. Louis, MO, USA) to the culture medium. The procoagulant activity of the cultured cells was evaluated using rabbit plasma [7]; lactate levels in the culture media were also examined using a lactate oxidase-based method; the values were normalized to the total cellular protein content. The methods used for the measurement of the mRNA and protein expression levels and procoagulant activity of the cultured cells are described below. This study was approved by the research ethics committee at the University of Miyazaki (O-0272) and all participants provided their written informed consent.

### RNA extraction from cultured macrophages

PBMC-derived macrophages were cultured in 12-well plates, washed with PBS, and lysed with 0.5 mL of TRIzol (Invitrogen, Waltham, MA, US). Total RNAs were extracted using the RNA Mini Kit (Invitrogen, Waltham, MA, US) and quantified using the NanoDrop 1000 spectrophotometer (Thermo Scientific, Rockford, IL, USA).

### Quantitative real-time reverse transcription polymerase chain reaction

Single-stranded complementary DNA (cDNA) was prepared from the extracted RNAs using the PrimeScript RT reagent kit (Perfect Real Time, Takara Bio, Shiga, Japan) and used for real-time polymerase chain reaction (PCR).

PCR was performed using the LightCycler 96 System (Roche, Basel, Switzerland), SYBR Premix EX Taq II (Perfect Real Time, Takara Bio), and specific primers with the following sequences: β-actin, 5′-TGGCACCCAGCACAATGAA-3′ (forward) and 5′-TAAGTCATAGTCCGCCTAG-AAGCA-3′ (reverse); TF, 5′-TGACCTCACCGACGAGATTGTGAA-3′ (forward) and 5′-TCTGAATTGTTGGCTGTCCGAGGT-3′ (reverse); HK-II, 5′-CTAGGCTGAGCTGGCATT-GG-3′ (forward) and 5′-TAGGACAGAGGCGGGCTTTC-3′ (reverse). The gene expressions were normalized by β-actin expression.

### Protein expression analysis using an enzyme-linked immunosorbent assay

Total protein was extracted from cultured macrophages using Pierce RIPA Buffer (Thermo Fisher Scientific, Waltham, MA, US) containing 1% Halt Protease Inhibitor Cocktail (Thermo Fisher Scientific). The protein concentration was determined using the Pierce BCA Protein Assay Kit (Thermo Fisher Scientific). TF was quantified using the Quantikine Colorimetric Sandwich ELISA kit (R&D Systems, Minneapolis, MN, US) and normalized to the total protein concentration.

### Evaluation of the procoagulant activity of the cultured macrophages

The procoagulant activity of cultured macrophages was measured using the KC-1 Delta (Tcoag Ireland Ltd., Wicklow, Ireland) [7]. Macrophages on the bottom surface of 12-well plates were washed thrice with PBS and then incubated with trypsin-ethylenediaminetetraacetic acid at 37 °C for 2–5 min.

The cells were then harvested with PBS and centrifuged at 1,000 ×  *g* for 5 min to pellet cells. After PBS removal, the cells were resuspended in TSC buffer (50 mM Tris-HCl, pH 7.4, containing 100 mM NaCl, 10 mM sodium citrate, 0.02% sodium azide, and 10 mM CaCl_2_). Cell density was measured using Scepter (Merck Millipore, Billerica, MA, US) and standardized to the minimum concentration sample using TSC buffer. To measure the rabbit plasma clotting time, 100 μL of cell suspension was added to test tubes filled with 100 μL of rabbit plasma and 100 μL of CaCl_2_ (20 mmol/L), and the time until the test reagent clotted was recorded using KC-1 Delta. Thromborel S (Siemens Healthcare Diagnostics, Deerfield, IL, US) was used to induce clotting, and the clotting time values were used to construct a calibration curve for the representative rabbit plasma. The procoagulant activity of the macrophages was calculated as an arbitrary unit using the calibration curve. The TSC buffer alone did not cause plasma clotting, even after 900 s. This study was approved by the Animal Care and Use Committee of Miyazaki University (No. 2022-513).

### Statistical analysis

Data were analyzed using GraphPad Prism 8 software (GraphPad Software Inc., San Diego, CA, USA) and are presented as medians of individual values. Data were analyzed using an unpaired *t*-test or one-way analysis of variance when showing a normal distribution according to the Shapiro–Wilk test. Data that did not show a normal distribution were analyzed using the Mann–Whitney *U* or Kruskal–Wallis test. Statistical significance was set at p <  0.05.

## Results

### Human vulnerable coronary lesions showed higher expression of HK-II and TF

**Fig 1:**
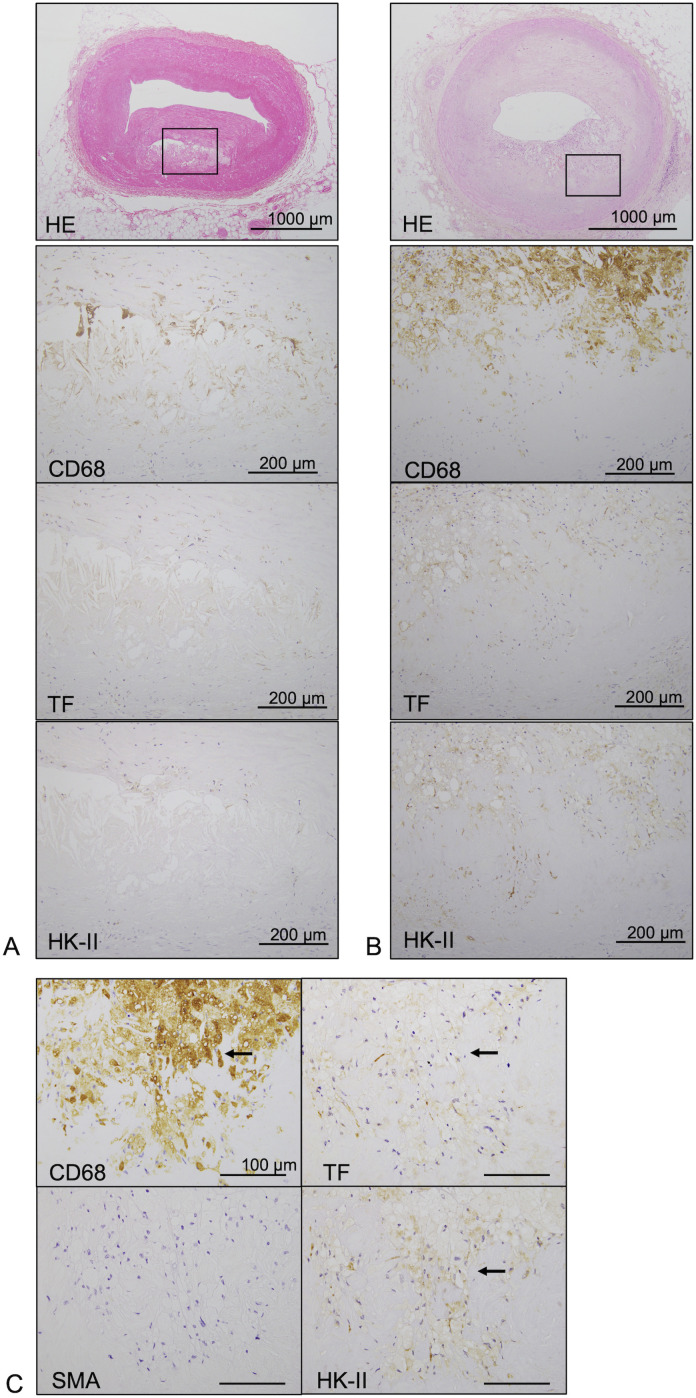
Expression of TF and HK-II in stable and vulnerable coronary atherosclerotic plaques. **(A)** Representative histological and immunohistochemical images of a fibrous cap atheroma (stable lesions). In the high-magnification images (square), the stable plaque shows a mild infiltrate of CD68-immunopositive cells around the necrotic core and modest expression of TF and HK-II. **(B)** Representative histological and immunohistochemical images of a thin-cap fibroatheroma (vulnerable lesions). In the high-magnification images (square), the vulnerable plaque shows the presence of CD68-immunopositive cells in the fibrous cap and necrotic core. TF- and HK-II-immunopositive cells are predominantly localized in macrophage-rich areas. **(C)** Representative immunohistochemical high-magnification images of a thin-cap fibroatheroma (vulnerable lesions). CD68-immunopositive cells are immunopositive for TF and HK-II (arrow) but not SMA.

**Fig 2:**
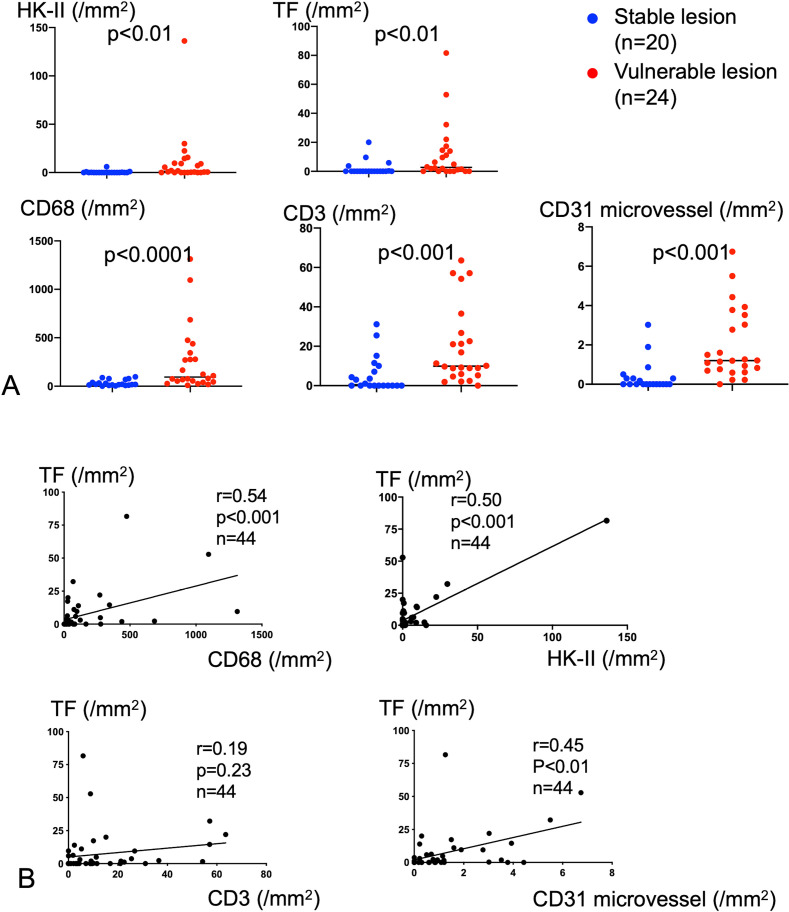
Immunohistochemical expression of HK-II, TF, and vascular cells in stable and vulnerable lesions of human coronary arteries. **(A)** HK-II, TF, CD68, CD31, and CD3 expression in stable and vulnerable human coronary lesions. Mann–Whitney *U* test. **(B)** Relationships among CD68, HK-II, CD3, CD31, and TF expression in the coronary artery. Spearman rank correlation analysis.

To determine the expression and localization of a hypoxic marker and the thrombogenic potential in coronary plaques, we immunohistochemically examined HK-II and TF expression in coronary artery tissue samples from autopsy cases. Forty-four coronary sections were obtained from 12 patients (Table 1). The coronary lesions included 20 stable lesions (33%) and 24 vulnerable lesions (67%). Fig 1A and 1B presents the findings for a stable fibroatheroma and a vulnerable thin-cap fibroatheroma, showing the formation of a necrotic core covered by a thin fibrous cap, respectively. Immunohistochemically, the stable plaque showed a mild infiltrate of CD68-immunopositive cells around the small necrotic core and modest expression of TF and HK-II (Fig 1A). In contrast, the vulnerable plaque showed a dense infiltrate of CD68-immunopositive cells in the plaque, and TF- and HK-II-immunopositive cells were predominantly localized in macrophage-rich areas (Fig 1B and 1C). Representative immunohistochemistry images for CD3 and CD31 of plaques are shown in S1 Fig. The number of cells immunopositive for HK-II, TF, CD68, and CD3 was significantly higher in vulnerable plaques than those in stable plaques. CD31-immunopositive microvessel density in vulnerable plaques was higher than in stable plaques (Fig 2A). The number of CD68- and HK-II-immunopositive cells and the microvessel density showed a significant positive correlation with the number of TF-immunopositive cells. No significant correlation was observed between the number of CD3- and TF-immunopositive cells (Fig 2B). In addition, the number of MMP9-immunopositive cells in vulnerable plaques was higher than in stable plaques. The number of MMP-9 immunopositive cells significantly correlated with the that of TF immunopositive cells in coronary plaques (S2 Fig).

**Table 1:** AHA type of coronary plaque histology from 12 autopsy cases.

Stable lesion (n = 20)		
Type I	3	(17%)
Type II	3	(25%)
Type III	4	(8%)
Type IV	4	(0%)
Type V without thin fibrous cap	6	(50%)
Vulnerable lesion (n = 24)		
Type V with thin fibrous cap	7	(29%)
Type VI	17	(71%)

We immunohistochemically examined the expression of iNOS (a marker of M1-like macrophages), MRC-1, and CD163 (markers of M2-like macrophages) in coronary stable and vulnerable plaques. M1-like and M2-like marker immunopositive cells were observed in stable and vulnerable plaques (S3A Fig for stable lesion, S3B Fig for vulnerable lesion). The number of iNOS immunopositive cells in vulnerable plaques was higher than that in stable plaques, while the number of MRC-1- and CD163-immunopositive cells tended to be higher than that of iNOS-immunopositive cells. The number of MRC-1- and CD163- immunopositive cells did not differ between stable and vulnerable plaques (S4 Fig).

We also determined whether coronary calcification affected plaque vulnerability and TF or HK-II expression in macrophages in coronary arteries. Among the histological types of calcification, fragment calcification was more frequent in vulnerable plaques (54%) than in stable lesions (0%, p <  0.0001) (Table S1). Most histological types of calcification did not affect TF or HK-II expression, while HK-II positive cell density was higher in coronary plaques with fragment calcification than those without (S5 Fig).

### TF- and HK-II-expressing macrophages in the culprit thrombi from ACS patients

To examine the presence or absence of TF- and HK-II-expressing cells in the culprit coronary thrombi at the onset of plaque rupture, we histologically assessed aspirated coronary thrombi with ruptured plaque components obtained from patients with ACS.

**Fig 3:**
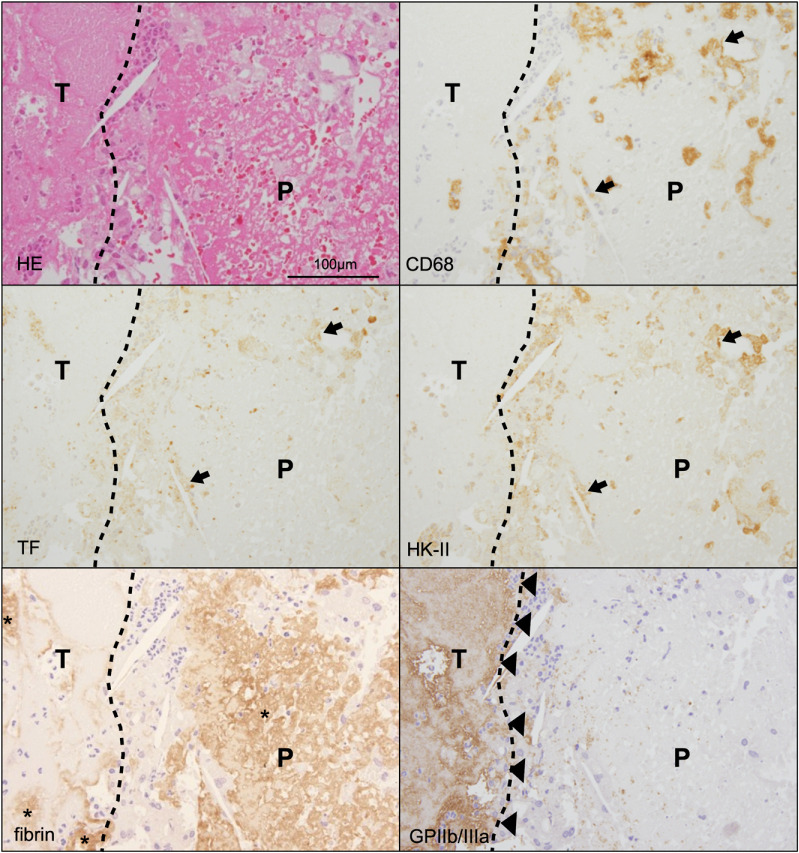
Representative histological and immunohistochemical images of aspirated thrombus with ruptured plaque in patients with ACS. The ruptured plaque and thrombus interface demonstrated the involvement of CD68-, HK-II-, and TF-triple immunopositive cells in fibrin and platelet aggregation (arrows). Fibrin formation is localized in the ruptured plaque and a part of the thrombus (asterisks). Platelet aggregation is observed on ruptured plaque (arrowheads). The dotted lines indicate the boundary between thrombus (T) and ruptured plaque (P).

Fig 3 shows the histological and immunohistochemical images of ruptured coronary plaques with thrombus formation in patients with ACS. Platelet aggregation was almost localized in thrombi. On the contrary, fibrin formation was observed in the thrombi and ruptured plaques. CD68-immunopositive macrophages and cholesterol clefts were involved in fibrin formation and platelet aggregation in all culprit lesions (n =  15, 100%). HK-II- and TF-immunopositive macrophages were surrounded by fibrin or aggregated platelets in all lesions (both, n =  15, 100%). SMA- or CD31-immunopositive cells were noted in 3 (20%) and 0 (0%) samples of the ruptured plaques, respectively. No SMA- or CD31-immunopositive cells showed contact with the coronary thrombi (Table 2). On expression of macrophage polarization markers, the frequencies of iNOS, MRC-1, and CD163 immunopositive cells in coronary ruptured plaque with thrombus were 80%, 53%, and 100%, respectively. The frequencies of contact of iNOS, MRC-1, and CD163 immunopositive cells with aspirated coronary thrombi were 67%, 47%, and 100%, respectively (Table 2). Representative immunohistochemical images of iNOS, MRC-1, and CD163 are in S6 Fig.

**Table 2:** Presence of immunopositive cells and their contact with thrombi in aspirated samples from coronary arteries (n = 15).

	Presence		Contact	
CD68	15	(100%)	15	(100%)
Hexokinase II	15	(100%)	15	(100%)
Tissue factor	15	(100%)	15	(100%)
Smooth muscle actin	3	(20%)	0	(0%)
CD31	0	(0%)	0	(0%)
iNOS	12	(80%)	10	(67%)
MRC-1	8	(53%)	7	(47%)
CD163	15	(100%)	15	(100%)

### Hypoxia further augmented TF expression and procoagulant activity in TNFα/INFγ-stimulated macrophages

**Fig 4:**
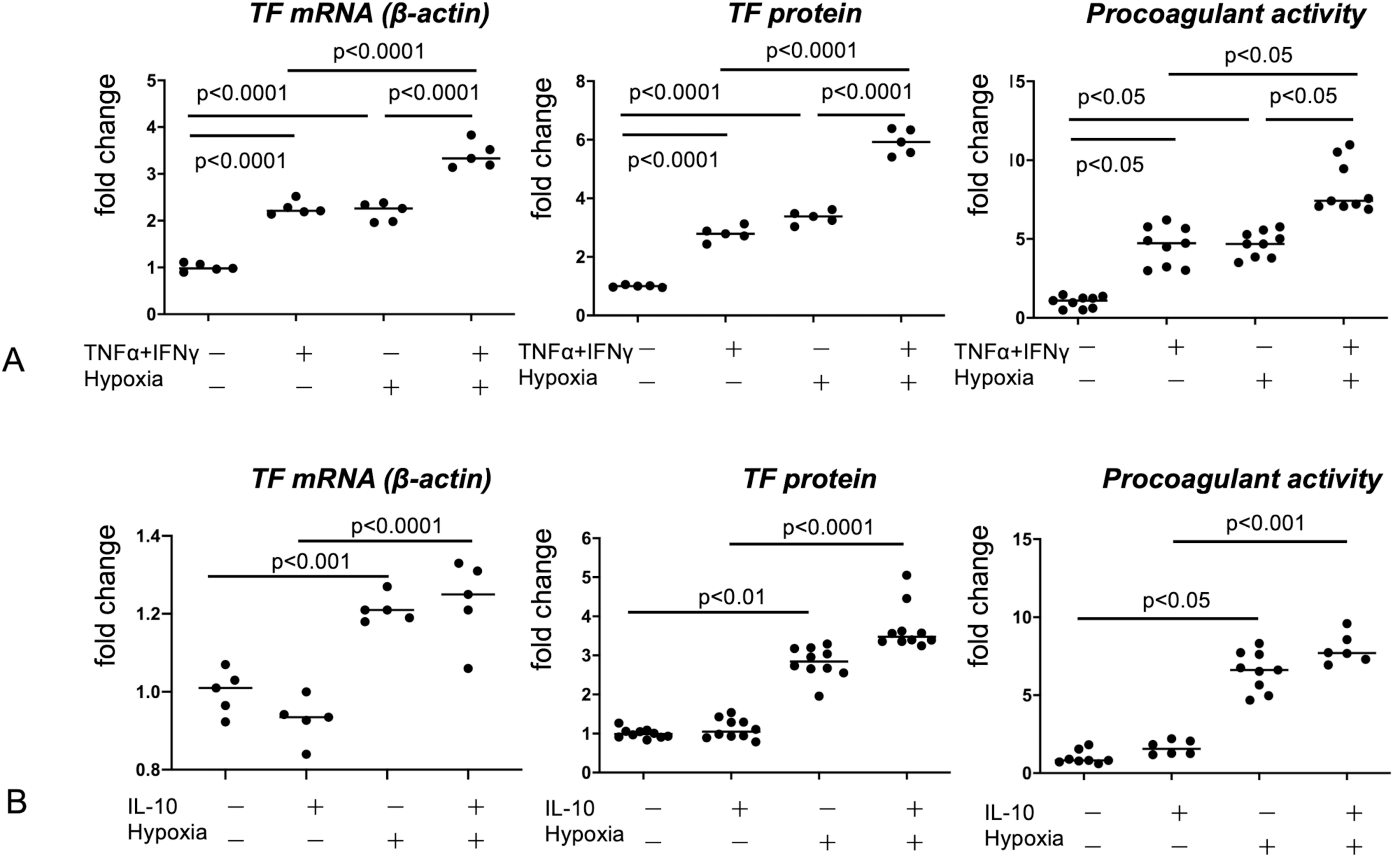
TF expression and procoagulant activity in PBMC-derived macrophages under inflammatory stimuli and hypoxia. PBMC-derived macrophages were stimulated by TNFα (10 mg/mL) and INF-γ (20 ng/mL), hypoxia (1% O2), or IL-10 (10 ng/mL) for 4 **h** (TF mRNA expression) or 24 **h** (TF protein expression and procoagulant activity). Data are expressed as fold changes in comparison with the nonstimulated control. One-way analysis of variance was performed with Sidak’s multiple-comparison tests (A, TF mRNA and TF protein; B, TF mRNA). Kruskal–Wallis test was performed with Dunn’s multiple-comparison test (A, procoagulant activity; B, TF protein, procoagulant activity).

To determine whether the combination of inflammatory stimuli and hypoxic conditions augmented thrombogenicity, we examined TF expression and procoagulant activity in PBMC-derived macrophages. TNFα/INF-γ stimulation or hypoxic conditions considerably increased TF mRNA, protein expression, and procoagulant activity. The combination of the TNFα/INF-γ stimulus and hypoxic conditions further augmented TF mRNA, protein expression, and procoagulant activity in comparison with the individual stimuli (Fig 4A). IL-10 stimulation did not affect TF mRNA and protein expression or procoagulant activity under normoxic and hypoxic conditions (Fig 4B).

### Hypoxia, not TNFα/INF-γ, augmented HK-II mRNA expression and glycolysis in macrophages

**Fig 5:**
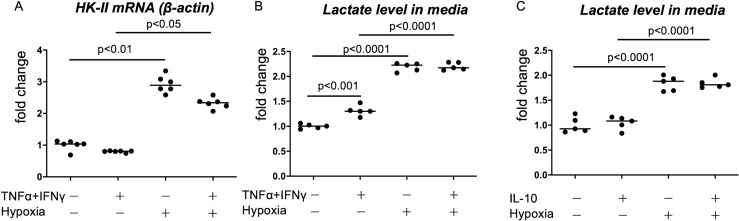
HK-II expression and lactate production in PBMC-derived macrophages under inflammatory stimuli and hypoxia. PBMC-derived macrophages were stimulated by TNFα (10 mg/mL) and INF-γ (20 ng/mL), hypoxia (1% O2), or IL-10 (10 ng/mL) for 4 **h** (A, HK-II mRNA expression) or 24 **h** (B, C, lactate production). Data are expressed as fold changes in comparison with the nonstimulated control. Kruskal–Wallis test was performed with Dunn’s multiple-comparison test (HK-II mRNA), and a one-way analysis of variance was performed with Sidak’s multiple-comparison test (lactate levels).

To determine whether HK-II expression and glycolysis in macrophages are influenced by TNFα/INF-γ stimulus, IL-10-stimulus, or hypoxic conditions, we examined the HK-II mRNA expression and lactate concentration in the culture media of PBMC-derived macrophages. Hypoxic stimuli upregulate HK-II mRNA expression. The TNFα/INF-γ stimulus did not affect HK-II mRNA expression under normoxic conditions but decreased the hypoxia-induced expression of HK-II mRNA (Fig 5A). The TNFα/INF-γ stimulus also increased lactate levels in culture media but did not affect lactate levels under hypoxic conditions (Fig 5B). The IL-10 stimulus did not affect lactate levels under normoxic or hypoxic conditions (Fig 5C).

### Inhibition of glycolysis reduced the augmentation of procoagulant activity induced by a combination of TNFα/INF-γ and hypoxia stimuli in macrophages

**Fig 6:**
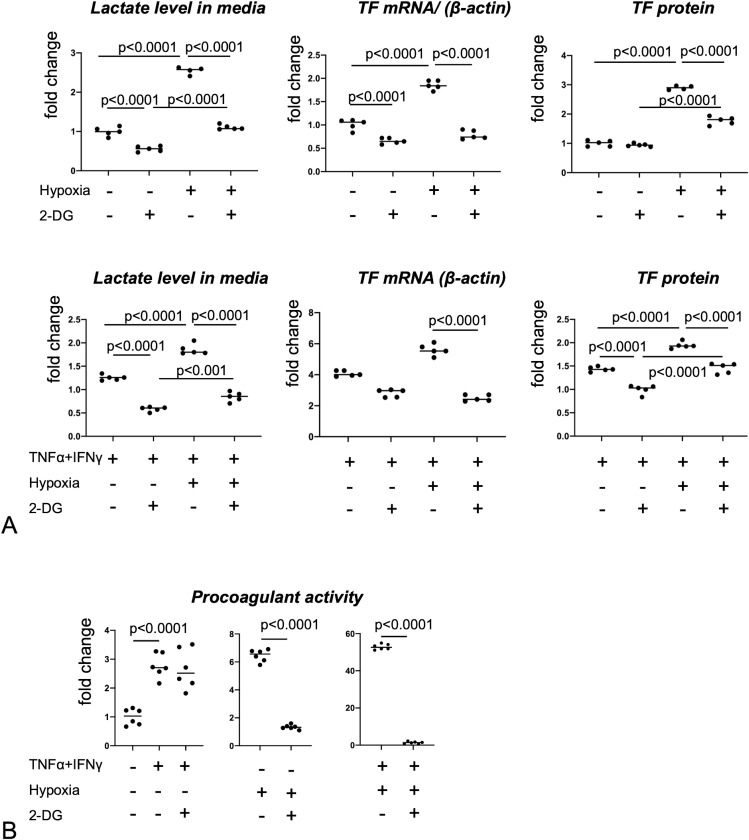
Lactate production, TF expression, and procoagulant activity in PBMC-derived macrophages under inflammatory stimuli, hypoxia, and inhibition of glycolysis. PBMC-derived macrophages were stimulated by TNFα (10 mg/mL) and INF-γ (20 ng/mL) and hypoxia (1% O_2_) for 4 **h** (TF mRNA expression) or 24 **h** (lactate production, TF protein expression, and procoagulant activity). Data are expressed as fold changes in comparison with the nonstimulated control. **(A)** Lactate production and TF expression in PBMC-derived macrophages under inflammatory stimuli, hypoxia, and inhibition of glycolysis. A one-way analysis of variance was performed with Sidak’s multiple-comparison test (lactate levels, TF mRNA upper, and TF proteins), and the Kruskal–Wallis test was performed with Dunn’s multiple-comparison test (TF mRNA lower). **(B)** Procoagulant activity in PBMC-derived macrophages under inflammatory stimuli, hypoxia, and inhibition of glycolysis. One-way analysis of variance was performed with Sidak’s multiple-comparison test or Student’s *t*-test.

The inhibition of glycolysis by 2-DG in cultured macrophages significantly reduced lactate levels in the culture medium under normoxic and hypoxic conditions (Fig 6A). Administration of 2-DG reduced the hypoxia-induced upregulation of TF mRNA and protein expression (Fig 6A). This administration also reduced TNFα/INF-γ-stimulated TF protein upregulation and combined TNFα/INF-γ- and hypoxia-stimulated TF mRNA and protein upregulation (Fig 6A).

The inhibition of glycolysis by 2-DG reduced the macrophage procoagulant activity enhanced by hypoxic conditions and combined TNFα/INF-γ stimulus and hypoxic conditions, but not by TNFα/INF-γ (Fig 6B).

## Discussion

In this study, we showed that unstable coronary plaques are rich in HK-II- and TF-expressing macrophages and that these HK-II- and TF-expressing macrophages are involved in coronary thrombus formation following plaque rupture. Hypoxic conditions augmented the TNFα/INF-γ-stimulated TF upregulation and procoagulant activity in cultured macrophages, and inhibition of glycolysis reduced the macrophage procoagulant activity enhanced by combined TNFα/INF-γ stimulus and hypoxia.

Most cases of ACS are caused by a coronary atherothrombus, which is initiated by disrupted atherosclerotic plaques. Rittersma et al. reported that all the aspirated coronary thrombus samples that included plaque components harbored macrophages in the plaque components [20]. A recent pathological study on the interface between the thrombus and the ruptured plaque revealed direct contact of aggregated platelets and fibrin with the exposed necrotic core and macrophages at the coronary ruptured sites [21]. Okuyama et al. also reported a positive relationship between coronary artery thrombus size and the expression of TF and HK-II in coronary artery plaques [7]. In the present study, TF- and HK-II-expressing macrophages were observed in all coronary ruptured plaques and were directly attached to the fibrin-rich thrombus (Fig 3). These findings suggest that plaque rupture leads to the exposure of TF-expressing hypoxic cells in the blood and initiates coronary thrombus formation, highlighting the association between TF expression and glycolysis in coronary macrophages. In contrast, no SMA- or CD31-immunopositive cells were observed to be in direct contact with the coronary thrombus (Table 2). Our data support the notion that hypoxic and TF-expressing macrophages, but not smooth muscle or vascular endothelial cells, play key roles in initiating coronary atherothrombus formation at rupture sites. M1- and M2-like macrophages were directly attached to the thrombus in the coronary thrombus aspiration samples (S6 Fig), although M1-like macrophages increased in vulnerable plaques in autopsy samples compared with stable plaques (S4 Fig). M1- and M2-like macrophages can express TF in coronary atherosclerotic lesions; cellular TF activity increased in M2-polarized compared with M1-polarized macrophages [7]. Therefore, M1- and M2-like macrophages may contribute to thrombus formation on ruptured plaques.

In human coronary arteries, the number of CD68-, CD3-, TF-, HK-II-, iNOS, and MMP9-immunopositive cells per mm^2^ area was significantly higher in vulnerable lesions than in stable lesions. In addition, macrophage content and HK-II expression were positively correlated with TF expression. These results are consistent with those of a previous study showing that the expression of HK-II in the carotid artery was enhanced in advanced atherosclerotic plaques compared to that in early atherosclerotic lesions [22]. However, in this study, not all macrophages expressed HK-II, and HK-II-expressing macrophages were localized in the relatively deep portion of the coronary plaques. In the carotid artery as well, the hypoxic area and nuclear localization of hypoxia-inducible factor -1a are observed at the center of advanced plaques [22,23]. Consistent with the hypoxic reaction, the microvessel density was higher in vulnerable than in stable plaques. In addition, T cells were abundant in vulnerable plaques. The pathological findings indicate the presence of combined inflammatory and hypoxic conditions in the human coronary artery and suggest that microenvironmental factors affect TF expression and thrombogenicity in plaque macrophages. TNFα, not hypoxia, induces MMP-9 expression in macrophages [24,25]. A relationship between TF and MMP-9 expression may be attributed to inflammatory conditions rather than hypoxia in coronary plaques.

Fragment calcification is localized on the edge of a large necrotic core [17]. In this study, fragment calcification was frequently observed in vulnerable plaques. HK-II expression was higher in coronary plaques with fragment calcification than those without. A mechanism of initiation and progression of calcification is associated with the death of SMCs and macrophages [26]. Hypoxia promotes macrophage necroptosis by activating hypoxia-inducible factor 1α via regulating microRNA-mediated ATP depletion [27]. Hypoxia in the large necrotic core may partly explain the increased expression of HK-II in plaque with fragment calcification.

Environmental factors affect TF expression in atherosclerotic plaques. TF expression in macrophages and SMCs is affected by Th1 and Th2 cytokines, hypoxia, C-reactive protein deposition, and oxidative stress [28,29]. Based on the fact that vulnerable lesions are rich in macrophages, T cells, TF-, and HK-II-expressing cells, and CD31-positive microvessels (Fig 2), and the fact that TF- and HK-II-expressing cells are directly involved in coronary thrombus formation (Fig 3), we focused on macrophage TF expression and thrombogenicity in response to the combination of inflammatory cytokines and hypoxia. As expected, TNFα/INF-γ stimulation or hypoxia augmented TF expression and procoagulant activity. Interestingly, combined stimulation of TNFα/INF-γ and hypoxia further increased TF expression and procoagulant activity. These findings suggest that macrophages in atherosclerotic lesions show heterogeneous thrombogenicity and that HK-II-expressing macrophages in large inflammatory plaques are more thrombogenic than macrophages under a single stimulus.

Cellular metabolism can also affect arterial thrombogenicity. Macrophages in human unstable coronary atherosclerotic lesions show upregulation of the expression of indoleamine 2,3-dioxygenase 1 (IDO1), a rate-limiting enzyme of the tryptophan kynurenine pathway, and IDO1 expression is associated with regulation of TF expression in macrophages [30]. An increase in the intracellular glutamine level downregulates TF expression and procoagulant activity in coronary artery SMCs [31]. In this study, lactate level in media was increased both by TNFα/INF-γ stimulation and by hypoxia. In contrast, HK-II mRNA expression was increased only by hypoxia but not by inflammatory stimuli (Fig 5). These results are consistent with the findings of Folco et al. [23], who reported that glucose uptake in macrophage, which reflects HK activity, was enhanced only by hypoxia, but not by inflammatory stimuli. The increase in lactate level under TNFα/INF-γ stimulation should be independent of HK-II. In a previous study using THP-1 cells, administration of 2-DG enhanced TF expression in M1- and M2-like polarized THP-1-derived macrophages [7]. THP-1, a human monocytic leukemia cell line, may differ in metabolism and response to stimuli from PBMC-derived macrophages. Fatty acid binding protein 3, a lipid metabolism-related protein, strongly upregulates GM-CSF-stimulated PBMC-derived macrophages but is not induced in phorbol 12-myristate 13-acetate-stimulated THP-1 macrophages [32]. IDO1 and IL-6 are upregulated in GM-CSF or GM-CSF/Th1 cytokine-stimulated PBMC-derived macrophages but are absent in phorbol 12-myristate 13-acetate stimulated THP-1 macrophages [33]. These differences between studies could be attributed to differences in cellular metabolism and responses to inflammatory stimuli. The effect of inflammatory cytokines on glycolysis seems small, especially under hypoxic conditions, since TNFα/IFNγ stimulation did not increase lactate levels in media but enhanced TF expression and procoagulant activity under hypoxic conditions. Therefore, inflammatory cytokines may enhance TF expression in macrophages via pathways other than glycolysis.

The present study has several limitations. First, although we observed TF- and HK-II-expressing macrophages in the thrombus at the site of plaque rupture, we could not determine whether these macrophages were stimulated by cytokines. Reliable immunohistochemical images of TNFα, IFNγ, and IL-10 could not be obtained in human coronary lesions of autopsy cases. This may be because the expression of each cytokine was not high enough to be detected or because of the time between death and fixation due to autopsy cases. Second, the fact that many of the autopsy cases evaluated in this study had ischemic heart disease might have resulted in a selection bias. We selected these cases since vulnerable plaques are rare in patients without ischemic heart disease. Nevertheless, minimizing the differences in clinical backgrounds between the stable and vulnerable plaque groups may be useful.

## Conclusion

In atherosclerotic microenvironments, TNFα/INF-γ stimulation and hypoxia may enhance TF expression and procoagulant activity in macrophages. Hypoxic macrophages may be exposed in the coronary circulation and initiate thrombus formation following plaque rupture. In addition, our data suggest a possible link between glucose metabolism and macrophage procoagulant activity.

## Supporting information

S1 Fig Expression of CD3 and CD31 in stable and vulnerable coronary atherosclerotic plaques.
(A) Representative histological and immunohistochemical images of a fibrous cap atheroma (stable lesions). In the high-magnification images (square), the stable plaque shows a mild infiltrate of CD3-immunopositive cells and few CD31-immunopositive microvessels around the necrotic core (arrow). (B) Representative histological and immunohistochemical images of a thin-cap fibroatheroma (vulnerable lesions). In the high-magnification images (square), the vulnerable plaque shows increased CD3-immunopositive cells and CD31-immunopositive microvessels at the shoulder lesion (arrows).
(TIF)

S2 Fig Expression of MMP9 in stable and vulnerable coronary atherosclerotic plaques.
(A) Representative histological and immunohistochemical images of a fibrous cap atheroma (stable lesions). In the high-magnification images (square), the stable plaque shows a mild infiltrate of MMP9-immunopositive cells around the necrotic core (arrow). (B) Representative histological and immunohistochemical images of a thin-cap fibroatheroma (vulnerable lesions). In the high-magnification images (square), the vulnerable plaque shows increased MMP9-immunopositive cells at the periphery of and in the necrotic core (arrows). (C) MMP-9-immunopositive cell densities in stable and vulnerable lesions. Mann–Whitney *U* test. (D) Relationships between MMP9 and TF expression in the coronary arteries. Spearman rank correlation analysis.
(TIF)

S3 Fig Expression of iNOS, MRC-1, and CD163 in stable and vulnerable coronary atherosclerotic plaques.
(A) Representative histological and immunohistochemical images of a fibrous cap atheroma (stable lesions). (B) Representative histological and immunohistochemical images of a thin-cap fibroatheroma (vulnerable lesions). M1-like macrophages (iNOS-positive) and M2-like macrophages (MRC-1- and CD163-positive) are present in stable (A) and vulnerable plaques (B) (arrows). iNOS immunopositive cells are more in the vulnerable lesion (B) than the stable lesion (A). High-magnification images focus on the squared area.
(TIF)

S4 Fig iNOS-, MRC-1-, and CD163-immunopositive cell densities in stable and vulnerable coronary atherosclerotic plaques.
Mann–Whitney *U* test.
(TIF)

S5 Fig Expression of tissue factor (TF) and HK-II in coronary plaques with or without calcification.
TF and HK-II immunopositive cell densities in coronary plaques with or without micro/punctate calcification, fragment calcification, and sheet calcification. Mann–Whitney *U* test.
(TIF)

S6 Fig Macrophage polarization in aspirated coronary thrombus with ruptured plaque.
M1-polarized iNOS-immunopositive cells and M2-polarized MRC-1- and CD163-immunopositive cells are present in the ruptured plaque component and are in contact with the thrombus (arrows). The dotted line in the hematoxylin and eosin (HE) stain shows the borderline between the thrombus and plaque component.
(TIF)

S1 Table Characteristics of coronary calcification. (TIF)
