# Supplementary figures and images for: Inflammatory stimuli and hypoxia on atherosclerotic plaque thrombogenicity: Linking macrophage tissue factor and glycolysis
Source: PLoS One. 2025 Mar 4;20(3):e0316474. doi: 10.1371/journal.pone.0316474 (PMC11878908; doi:10.1371/journal.pone.0316474)

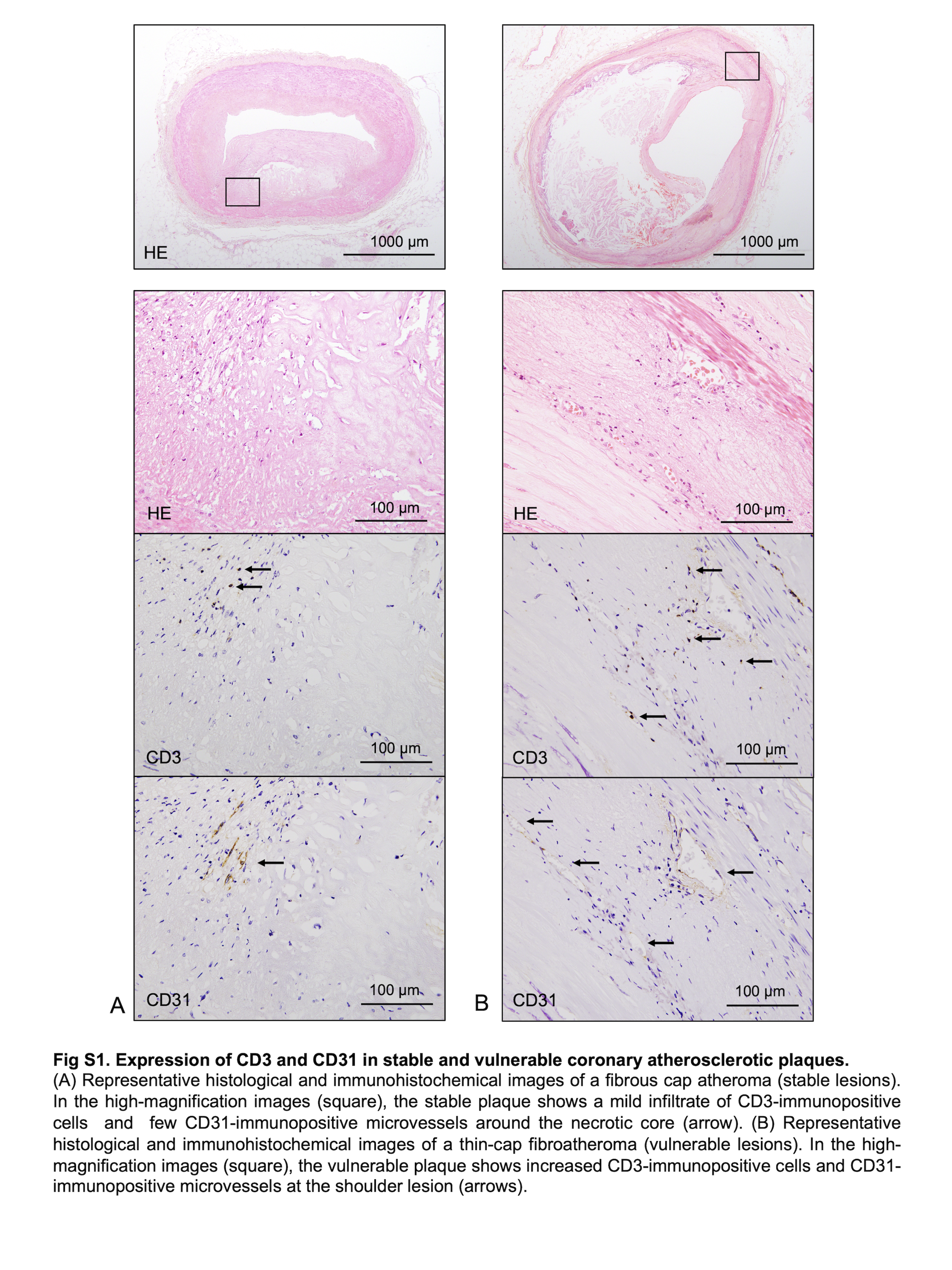

Supplement: S1 Fig — (A) Representative histological and immunohistochemical images of a fibrous cap atheroma (stable lesions). In the high-magnification images (square), the stable plaque shows a mild infiltrate of CD3-immunopositive cells and few CD31-immunopositive microvessels around the necrotic core (arrow). (B) Representative histological and immunohistochemical images of a thin-cap fibroatheroma (vulnerable lesions). In the high-magnification images (square), the vulnerable plaque shows increased CD3-immunopositive cells and CD31-immunopositive microvessels at the shoulder lesion (arrows). (TIF) [file pone.0316474.s001.tif]

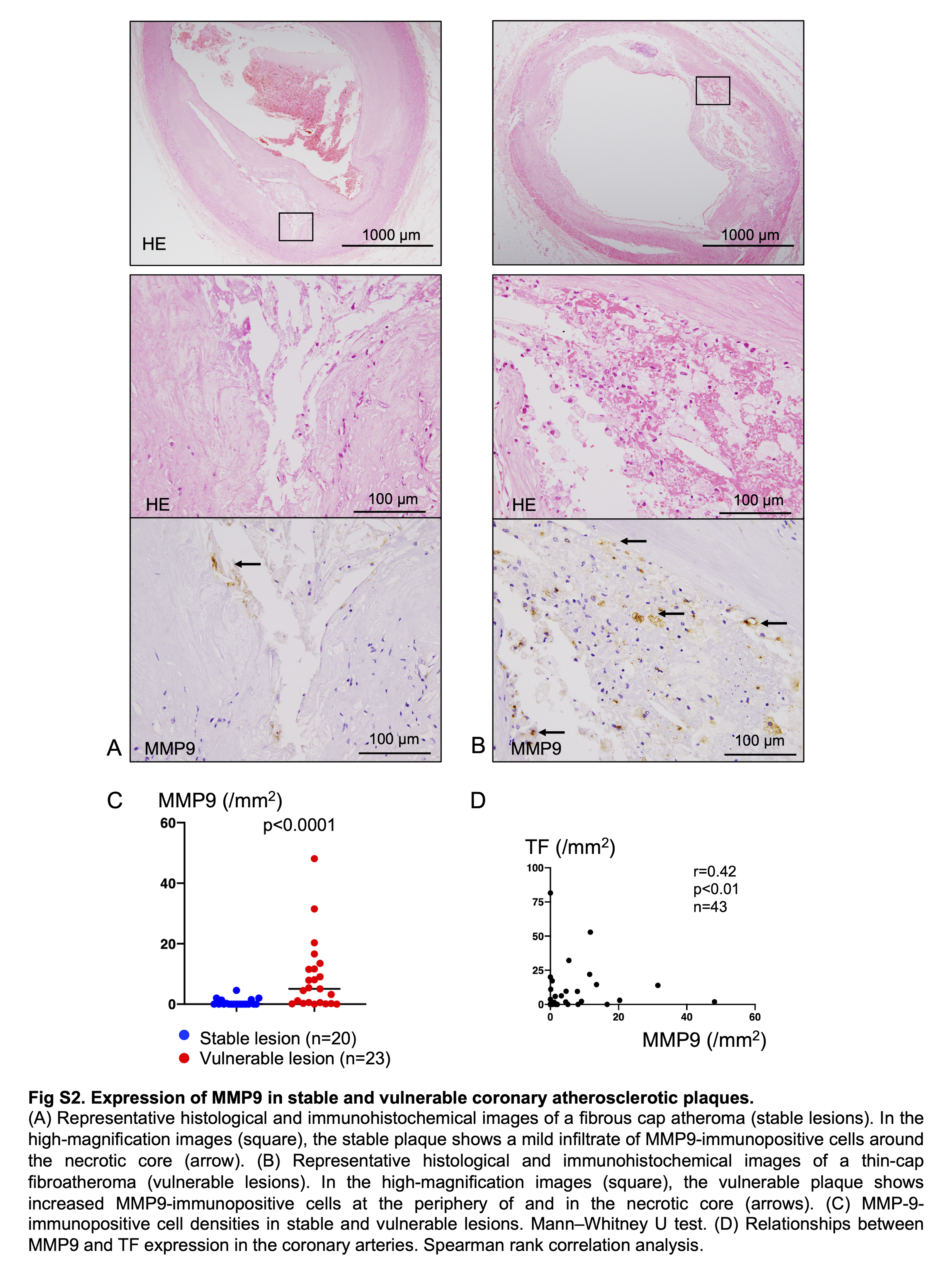

Supplement: S2 Fig — (A) Representative histological and immunohistochemical images of a fibrous cap atheroma (stable lesions). In the high-magnification images (square), the stable plaque shows a mild infiltrate of MMP9-immunopositive cells around the necrotic core (arrow). (B) Representative histological and immunohistochemical images of a thin-cap fibroatheroma (vulnerable lesions). In the high-magnification images (square), the vulnerable plaque shows increased MMP9-immunopositive cells at the periphery of and in the necrotic core (arrows). (C) MMP-9-immunopositive cell densities in stable and vulnerable lesions. Mann–Whitney U test. (D) Relationships between MMP9 and TF expression in the coronary arteries. Spearman rank correlation analysis. (TIF) [file pone.0316474.s002.tif]

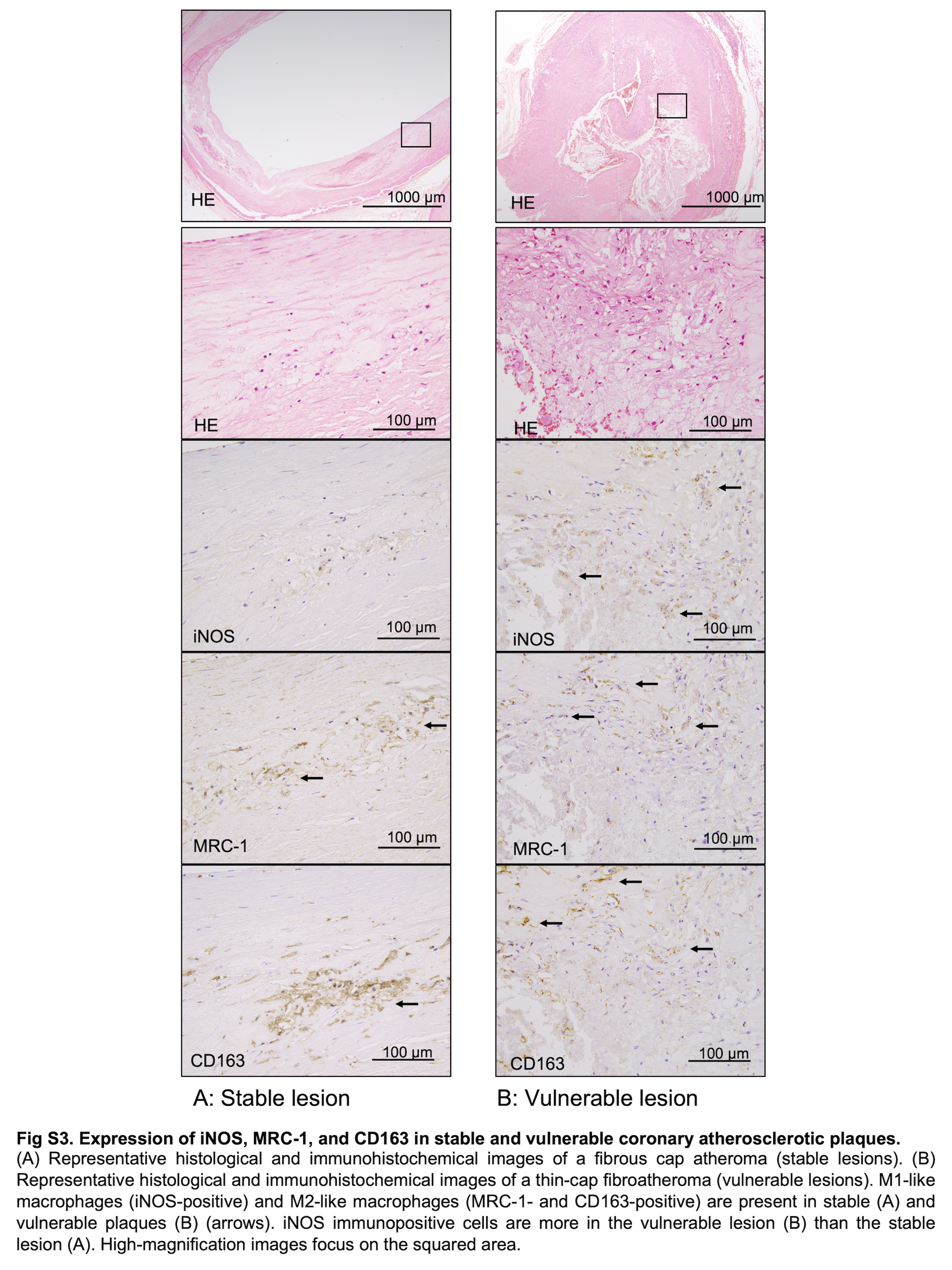

Supplement: S3 Fig — (A) Representative histological and immunohistochemical images of a fibrous cap atheroma (stable lesions). (B) Representative histological and immunohistochemical images of a thin-cap fibroatheroma (vulnerable lesions). M1-like macrophages (iNOS-positive) and M2-like macrophages (MRC-1- and CD163-positive) are present in stable (A) and vulnerable plaques (B) (arrows). iNOS immunopositive cells are more in the vulnerable lesion (B) than the stable lesion (A). High-magnification images focus on the squared area. (TIF) [file pone.0316474.s003.tif]

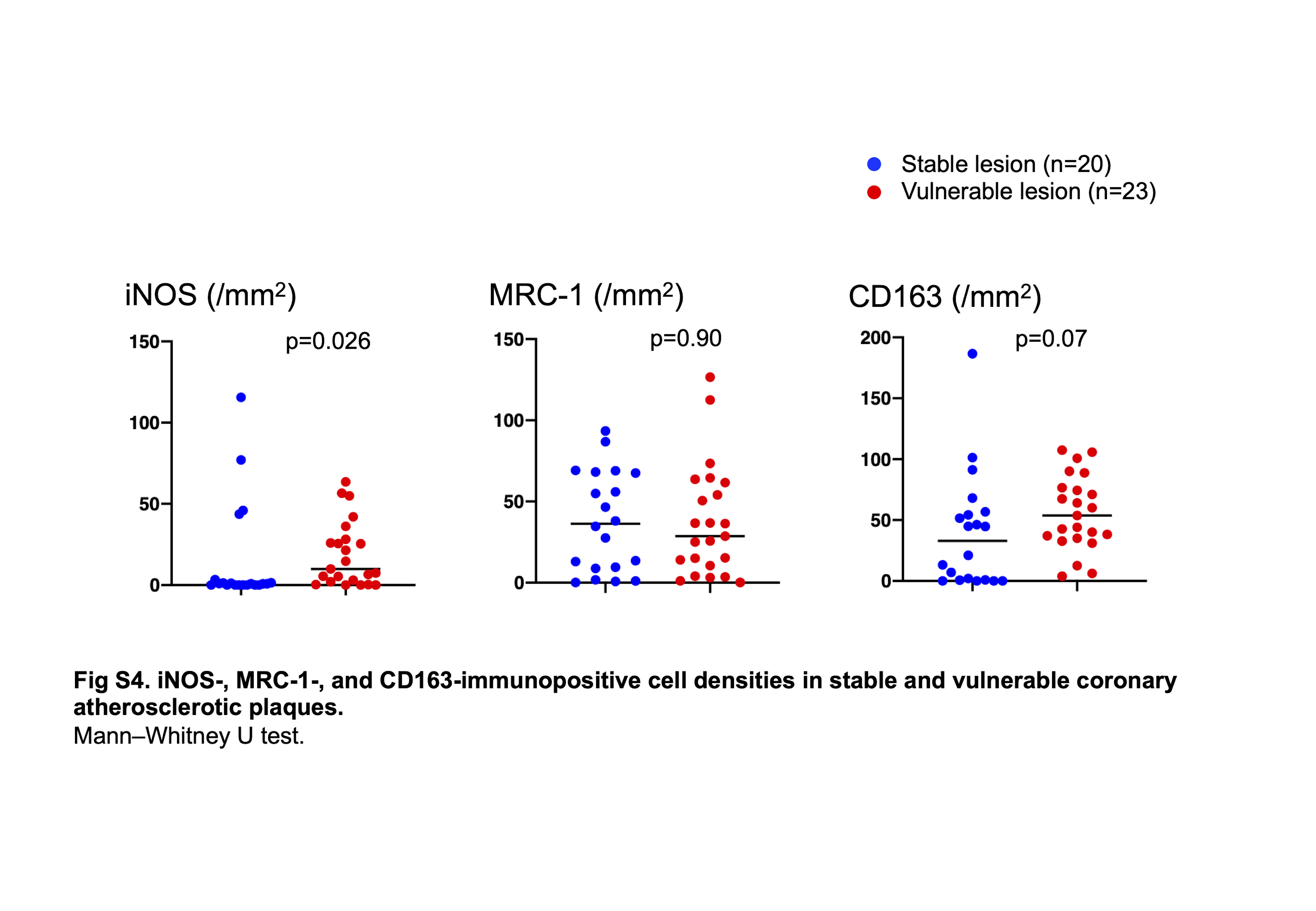

Supplement: S4 Fig — Mann–Whitney U test. (TIF) [file pone.0316474.s004.tif]

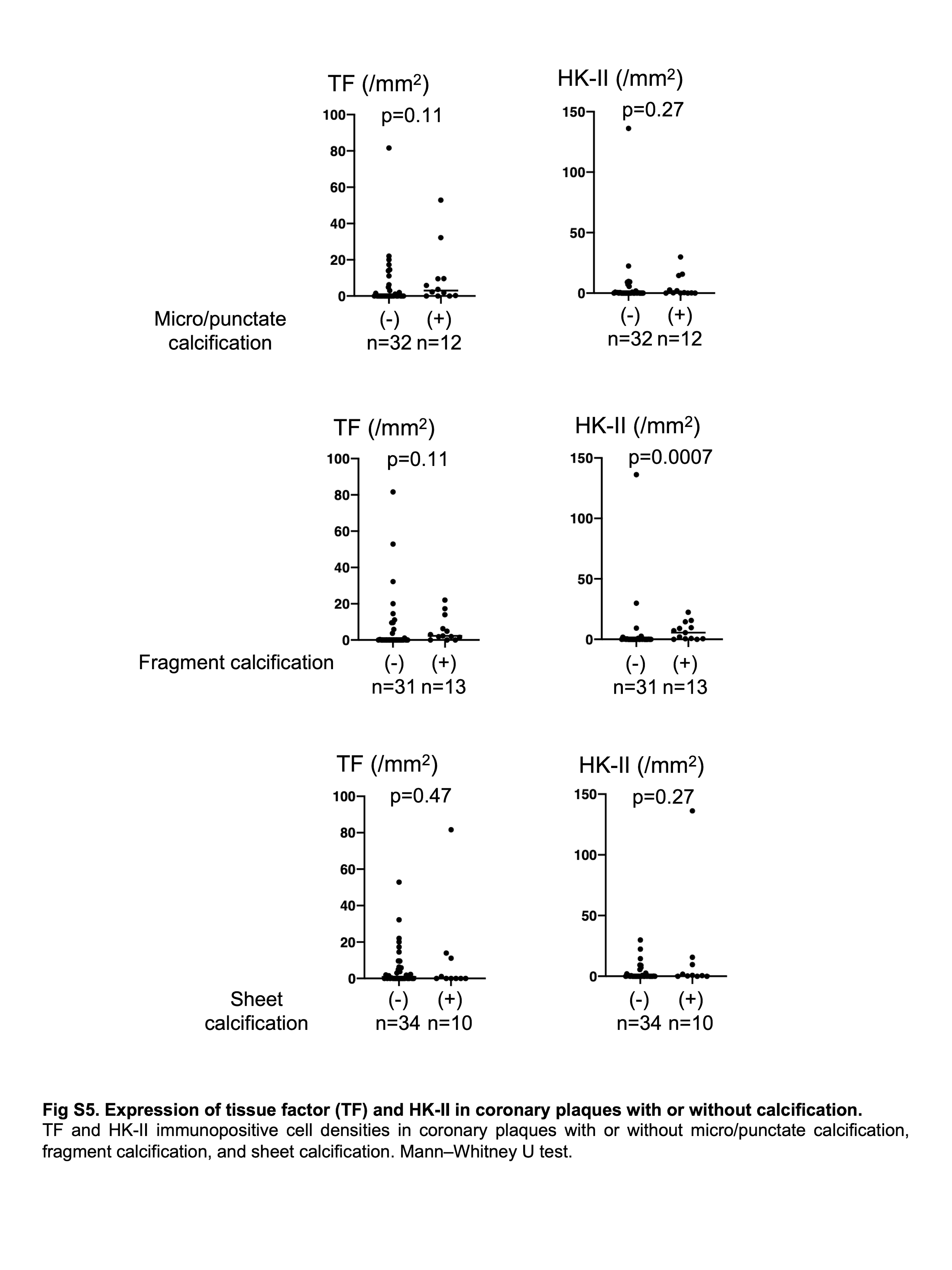

Supplement: S5 Fig — TF and HK-II immunopositive cell densities in coronary plaques with or without micro/punctate calcification, fragment calcification, and sheet calcification. Mann–Whitney U test. (TIF) [file pone.0316474.s005.tif]

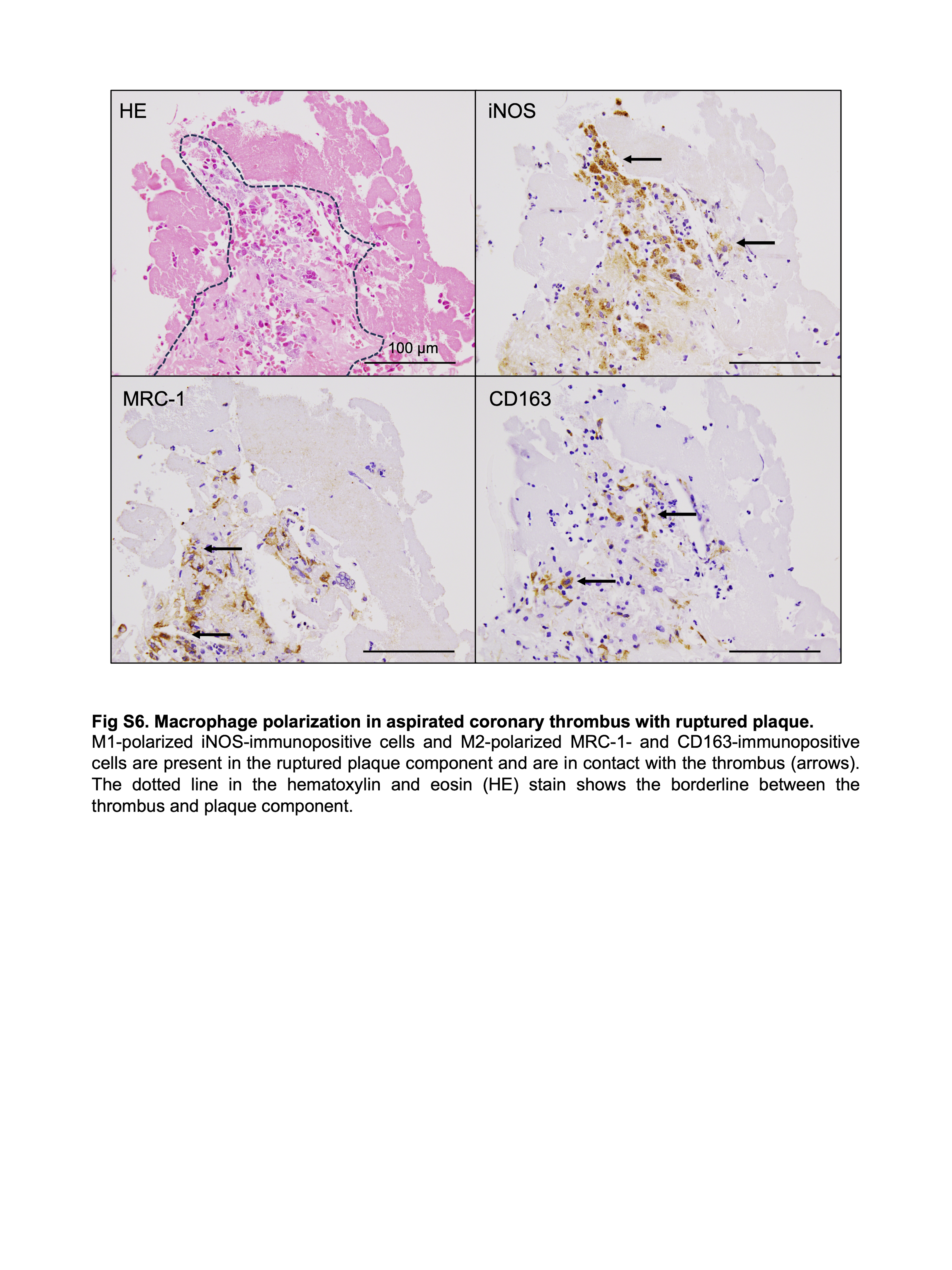

Supplement: S6 Fig — M1-polarized iNOS-immunopositive cells and M2-polarized MRC-1- and CD163-immunopositive cells are present in the ruptured plaque component and are in contact with the thrombus (arrows). The dotted line in the hematoxylin and eosin (HE) stain shows the borderline between the thrombus and plaque component. (TIF) [file pone.0316474.s006.tif]

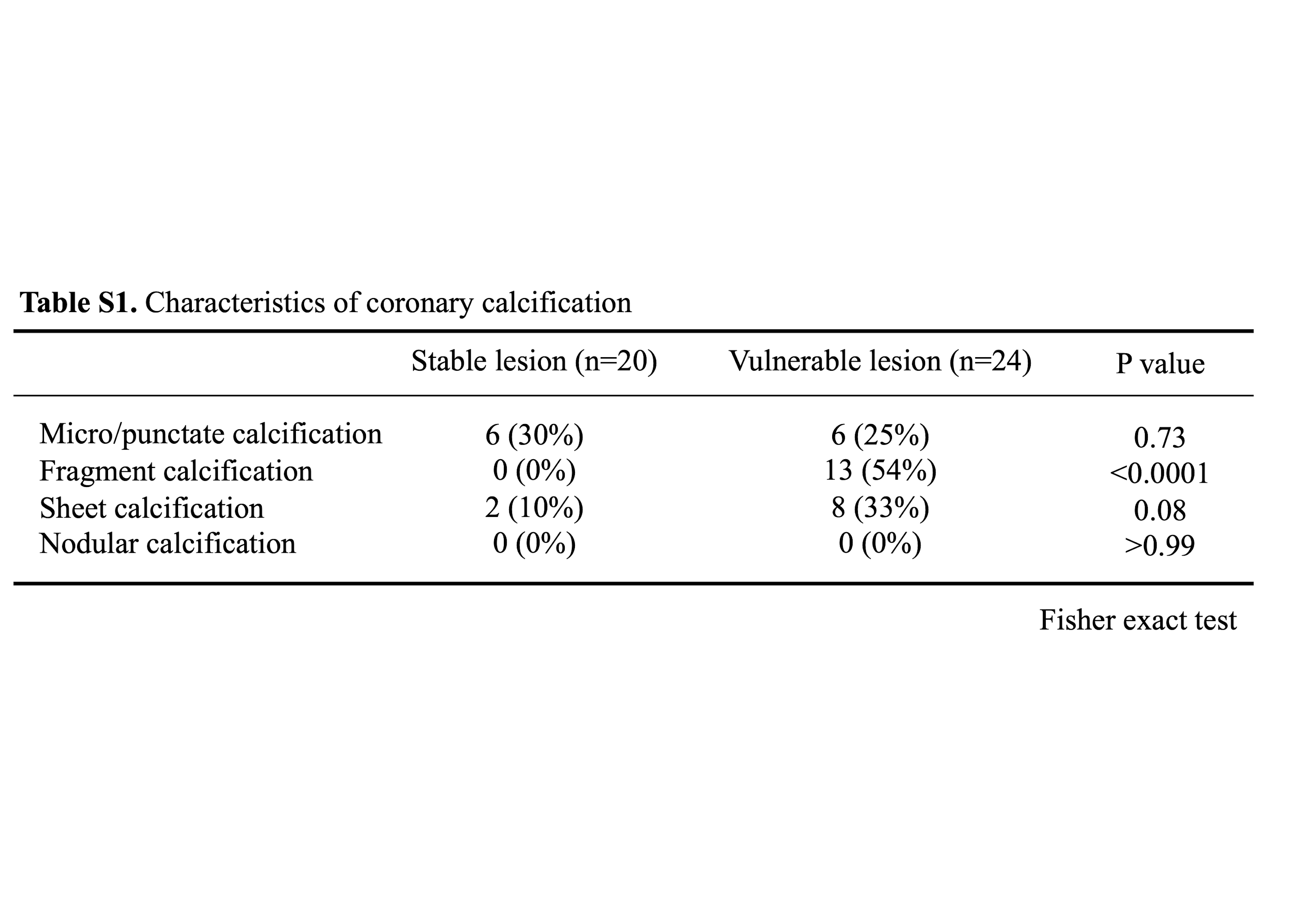

Supplement: S1 Table — (TIF) [file pone.0316474.s007.tif]
